# Conducting Phase I Trials During the SARS-Coronavirus-2 Outbreak: About Science and Care

**DOI:** 10.3389/fonc.2020.00926

**Published:** 2020-05-22

**Authors:** Angelo Dipasquale, Pasquale Persico, Elena Lorenzi, Monica Bertossi, Armando Santoro, Matteo Simonelli

**Affiliations:** ^1^Department of Biomedical Sciences, Humanitas University, Pieve Emanuele, Italy; ^2^Humanitas Cancer Center, Humanitas Clinical and Research Center—IRCCS, Rozzano, Italy

**Keywords:** Phase I (drug development), COVI-19, SARS - CoV-2, Cancer, clinical trial

It was December 2019 when severe acute respiratory syndrome coronavirus 2 (SARS-CoV-2) was identified for the first time in Wuhan, China. Since then, the outbreak has quickly evolved into a global pandemic with disruptive health, social, and economic consequences. As of April 20, 2020, the World Health Organization (WHO) reported 2.314.621 cases and 157.847 deaths across all continents, with exponential spread in Europe and now the United States (US) (1). Italy has been hurt dramatically, being the center of the pandemic for a while and still representing the second country after the US for number of victims (1, 2). To limit the spread of the infection across the population, a global lockdown has been set by the central government of Italy since the beginning of March. In this complex scenario, the Italian cancer community is facing many arising and tough challenges. Cancer patients have always been considered more susceptible to viral infections because of their own immunosuppression and, at least in theory, are at major risk of developing severe complications of the coronavirus disease (COVID-19), including adult respiratory distress syndrome (ARDS), leading to intensive care unit admission and even death. The first clinical reports from China seem to confirm these hypotheses, suggesting that patients with cancer are more likely to develop SARS-CoV-2 infection and have worse outcomes (3, 4). However, given the limited number of cases and significant biases, the conclusions of these studies are questionable, and the real impact of COVID19 on the cancer population is far from established.

We have experienced firsthand the dramatic impact of this pandemic, since our Cancer Center is located in the most affected region of Italy, Lombardy, and is part of an academic, clinical, and research hospital with its own emergency room admitting SARS-CoV-2-positive subjects on a daily basis. To face this unprecedented massive wave, the Humanitas Hospital has completely revolutionized its own organization in just a few days, redefining spaces and reallocating medical resources. Seven COVID-19 isolation wards have been created, accounting for over 250 beds, with more than 1,000 patients admitted so far, while all clinical activities in the oncology, cardiology, and stroke units have been temporarily suspended.

We all work in the phase I unit of our institution, a referring early-drug development hub evaluating and treating patients from all over the country. In the drug development program, phase I represents the connection point between preclinical research and clinical application of novel anticancer treatments. The main objective of phase I trials still remains the evaluation of safety, though the advent of significantly active new agents such as targeted therapies and immunotherapeutics in recent years means that participating in such studies undoubtedly represents a potentially effective therapeutic option (5–7). Conducting phase I trials has always been challenging for several reasons: the large number of complex protocol-scheduled procedures, such as blood and tissue sample collection, the need for multiple and repeated trips to the hospital, constant monitoring of adverse events, and interminable meetings with sponsors and other involved centers to share clinical information.

According to Hanna et al. (8), assessing the priority of indications for cancer care during this pandemic should carefully weigh the expected benefit, negative consequences of treatment delay and interruption, ability to safely deliver oncological services, and patients' willingness. In our opinion, recruitment into phase I trials retains a relevant place in the priority scale of cancer therapies, including during this emergency. This is why we decided to continue our activity even after the COVID-19 outbreak started impacting our hospital severely and some of us were relocated to the new dedicated COVID wards.

As we write, in northern Italy, the peak of the infection curve has just been passed, and the extraordinary pressure on hospitals is progressively reducing. With the storm seeming to be moving away toward the horizon, for our phase I unit team, it is the right time to take a first balanced look and reflect on the choices made. We generally adhered to the recommendations on the conduct of clinical trials issued by our national regulatory agency (9) while still making some specific considerations driven case-by-case by particular clinical scenarios and the risk to benefit ratio. First of all, we recommended our patients to strictly follow the rules for correct behavior, such as maintaining social distance, wearing a mask, and frequent hand-washing. To reduce the number of visits and to quieten anxiety about coming to a COVID-19 converted structure in the center of the pandemic, we allowed patients to perform control blood exams in the closest accredited facilities. We implemented phone call or video contacts for all scheduled visits considered not clinically urgent. Whenever possible, we strived to preserve PK sampling, at least during the first cycle of therapy. Protocol-scheduled on-treatment biopsies for pharmacodynamics purposes were avoided, and we discussed with sponsors the option of not performing screening biopsies if fresh or frozen tissues were still available. We delivered oral agents for two or more cycles (out of the first one) for patients in good and stable clinical condition. Experimental therapies were given in rooms with at least 1.5 meters of distance between seats or beds, with caregivers not permitted entry. Finally, we rescheduled several radiological tumor assessments according to protocol windows because of the significant need for CT scans in the Emergency Department (ED).

Beyond these measures, we also set up a specific path to triage phase I patients and to prevent contagion between patients and staff. At screening and before any hospitalization, all subjects underwent nasopharyngeal swab to rule out SARS-CoV-2 infection. In case of fever or respiratory symptoms during the scheduled appointment, we arranged an isolated route to ED, where patients underwent blood sample analysis, nasopharyngeal swab, and chest CT scan. For positive patients, hospitalization in a COVID-19-dedicated ward was consequently predisposed. Patients with a suggestive radiological picture who tested negative underwent a bronco-alveolar lavage (BAL) to rule the infection out definitively.

Phase I unit oncologists should play a key role in the multidisciplinary management of on-trial patients during an eventual hospitalization, supporting pulmonologists, and other specialists in daily clinical decision making. Particular attention should be paid to possible interactions between experimental agents and antivirals or chloroquine derivates in terms of pharmacokinetics modifications, cardiotoxicity (e.g., QTc prolongation), and liver toxicity. To obtain a correct differential diagnosis, we always have to keep in mind the potential overlap between immune-related adverse events (e.g., pneumonia, diarrhea, transaminase elevations) and some clinical pictures of COVID-19 as well as toxicities from drugs used to treat the infection. Considering its well-recognized negative impact on immunotherapy and being a potential exclusion criterion from many clinical trials, we should advise caution about the empirical use of steroids (e.g., methylprednisolone) for respiratory symptoms, which is to date not suggested by any COVID-19 guidelines (10–13). Indeed, we should help our colleagues in defining the prognosis of our patients and, accordingly, the indication for invasive respiratory support and resuscitation. Last but not least, given their denial of access to the COVID-19 wards, we should take charge of communication with the close relatives, keeping them constantly informed about the patient's clinical condition and course of hospitalization.

From the end of February to date, a total of 35 patients enrolled in phase I clinical trials continued to receive their experimental treatments, whether through intravenous, subcutaneous, or oral drugs. Though six industry-sponsored trials were placed on hold due to the COVID-19 pandemic, in this timeframe, we were able to screen 23 new patients, of which 17 were recruited into five different clinical trials. Moreover, we discussed with the sponsor and finally got the chance to include in an anti-PD-1 immunotherapy trial, formally put on hold, two patients affected by microsatellite-instable (MSI) tumors (endometrial and ovarian cancer) and without any other therapeutic option available.

To date, five patients enrolled in a phase I clinical trial were admitted to ED because of a suspicious clinical picture but tested negative for SARS-CoV-2 infection. The only on-trial case that tested positive so far was an entirely casual finding. This patient had a recent diagnosis of PD-L1 highly expressed metastatic lung adenocarcinoma, for which a first-line combination treatment of immunotherapy plus a multi-target tyrosine kinase receptor inhibitor was started. She was admitted because of a pathological vertebral fracture causing severe low-back pain and only collaterally was found to be infected by SARS-CoV-2. Since the chest CT scan showed a malignant pleural effusion without evidence of interstitial pneumonia, after discussion with a pulmonologist, thoracic surgeons, and anesthesiologists, we decided to perform a thoracoscopic pleural talcage, hoping to restart the experimental treatment after the resolution of infection.

It is truly hard to predict when the pandemic will end and what it will leave behind. In this complex and dynamic scenario, where the coexistence with SARS-CoV-2 seems to be unavoidable, at least until the arrival of a vaccine or effective antiviral drugs, we cannot reduce the intensity of care for cancer patients. Our experience suggests that the prosecution of phase I clinical trials is feasible and safe, even in hospitals admitting COVID-19 patients. All our efforts must be focused on implementing the complete separation of paths and the safety procedures required to protect patients and medical staff. The most important thing in these hard times is undoubtedly *sharing*, and we feel that others can benefit from our experience. It is not only a matter of science but almost a task to care.

## Author Contributions

All authors equally contributed to the conception and drafting of the present manuscript.

## Conflict of Interest

The authors declare that the research was conducted in the absence of any commercial or financial relationships that could be construed as a potential conflict of interest.
